# Modeling the emergence of multi-protein dynamic structures by principles of self-organization through the use of 3DSpi, a multi-agent-based software

**DOI:** 10.1186/1471-2105-6-228

**Published:** 2005-09-19

**Authors:** Hédi Soula, Céline Robardet, François Perrin, Sébastien Gripon, Guillaume Beslon, Olivier Gandrillon

**Affiliations:** 1 Laboratoire de Productique et d'Informatique des Systèmes Manufacturiers, Institut National des Sciences Appliquées de Lyon, Villeurbanne, France; 2 Centre de Génétique Moléculaire et Cellulaire CNRS UMR 5534; Université Claude Bernard Lyon 1, Villeurbanne, France

## Abstract

**Background:**

There is an increasing need for computer-generated models that can be used for explaining the emergence and predicting the behavior of multi-protein dynamic structures in cells. Multi-agent systems (MAS) have been proposed as good candidates to achieve this goal.

**Results:**

We have created 3DSpi, a multi-agent based software that we used to explore the generation of multi-protein dynamic structures. Being based on a very restricted set of parameters, it is perfectly suited for exploring the minimal set of rules needed to generate large multi-protein structures. It can therefore be used to test the hypothesis that such structures are formed and maintained by principles of self-organization. We observed that multi-protein structures emerge and that the system behavior is very robust, in terms of the number and size of the structures generated. Furthermore, the generated structures very closely mimic spatial organization of real life multi-protein structures.

**Conclusion:**

The behavior of 3DSpi confirms the considerable potential of MAS for modeling subcellular structures. It demonstrates that robust multi-protein structures can emerge using a restricted set of parameters and allows the exploration of the dynamics of such structures. A number of easy-to-implement modifications should make 3DSpi the virtual simulator of choice for scientists wishing to explore how topology interacts with time, to regulate the function of interacting proteins in living cells.

## Background

The possibility of probing the biophysical properties of fluorescent proteins in intact living cells by using confocal microscopy has led to a major step forward in contemporary biology. This technical advance has allowed biologists to obtain a new perception of different biological structures. Most of those structures were shown to be highly dynamic, to an extent that was previously unanticipated. This was shown to be especially important in studies of the nuclear architecture [1]. One important lesson from these studies is that, although various nuclear structures (including speckles, the nucleolus and various nuclear bodies) appear to be stable, their components are permanently engaged in an extraordinarily dynamic process: proteins are exchanged between nuclear structures and the nucleoplasm at a rate that makes the stability of the structures really astonishing. It has therefore been proposed that multi-protein dynamic structures are formed and maintained by principles of self-organization [1]. However, this provocative and speculative model raises the question of the stability of the nuclear structures: how do such structures reconcile the extensive material exchange with their environment *and *the global stability that we observe at a macroscopic level? Computer-based simulations can help to answer this question. If the self-organization hypothesis is true, then one should be able to virtually reconstruct computer-based model structures using a very restricted set of simple local interaction rules.

Most of the existing "virtual cell" models use an averaging behavior hypothesis. In this case, the overall phenomenon is a consequence of the mean behavior of an "average protein" inferred from those of a large number of single proteins. This assumption is inadequate in one or both of the following cases:

1. First when the number of molecules is too low to be correctly approximated by an average behavior [2]. For example, this is the case for transcriptional events that are increasingly being recognized as intrinsically stochastic events, mostly because the number of transcription factors is low [3].

2. Second, when the structures modeled have a strong spatial component. This will obviously be the case for the nuclear structures described above.

It has been proposed that multi-agent systems (MAS)-based modeling could provide a superior approach in those two contexts [4-9]. The study of MAS focuses on systems in which many "intelligent" agents interact with each other [10]. The agents are considered to be autonomous entities, such as software programs or robots. Their interactions can be either cooperative or selfish. That is, the agents can share a common goal (e.g. an ant colony), or they can pursue their own interests, as in free market models [11]. Most importantly, agents can be purposeless, i.e. they can be endowed with a very limited and simple set of rules. MAS have been successfully applied to various domains, including the popular boids, which mimick the structured motion of a flock of birds [12,13]. Such a use of artificial life to create bottom-up models of the real world follows from the realization from the Conway's life game [14], that simple rules can generate complex patterns.

We therefore decided to explore the MAS potential for modeling simple nuclear structures such as nuclear bodies, or speckles [1]. We demonstrate here that 3DSpi, a MAS-based software, is capable of explaining the emergence and predicting the behavior of cellular multi-protein dynamic structures.

## Implementation

### Program

3DSpi is built upon two libraries. The first one is called the Open Dynamic Engine (OpenDE or ODE) and is coding for the dynamic interactions. The second is the multi-agent framework called OpenSPEAR (Open Simulation of Physical Environment for Agent Research) and is has been developed specifically by us.

### Particle model

The program generates an environment which rules all the interactions between agents. Agents can be either single proteins or groups of proteins clustered together. These agents are considered here as solid 3-dimensional material with dynamic, collision and kinetic rules. All agents are animated by a random motion: at each time step (which corresponds to 1 ms) each agent is endowed with a random force and torque (rotation). The movement is then solved as well as collision (if any) for all agents sequentially.

At the beginning of a simulation, proteins are seeded at the intersection points of a grid (protein types are randomly chosen according to predefined proportions). In the experiments discussed here, this grid is a 6 × 6 × 6 grid and 3DSPi is therefore seeded with 216 particles (one at each intersection point). However, the size of the grid can be modified in order to seed the program with less or more proteins depending on the simulation purpose. Please note that this grid is used only for random seeding purposes and is not used again once the simulation has started.

During the simulations, agents are endowed with a random movement that they will pursue until they hit an obstacle, either the inner face of the nuclear membrane or another protein. In the case where they hit the inner face of the nuclear membrane, their speed is reduced to zero (this is called a soft shock). Since the protein is still animated with a brownian motion, it does not stay indefinitely close to the membrane. In the case where they hit another protein OpenDE is used to compute the resulting movements. Moreover, when a collision occurs, each of the involved agents computes whether it will stick and then at each of the following time steps whether it will stay stuck or not (see Coefficient of Stickiness in the "Parameters" paragraph).

In order to evaluate the dynamic structures that emerge, we needed to introduce a slightly different algorithm. Once two proteins are stuck, we create a meta-structure composed of both proteins. This is done recursively until all proteins composing one structure are within this meta-structure. We would like to stress that the goal of this meta-structure is strictly to compute the number of structures. It has no influence on the behavior of the system whatsoever.

### Parameters

In order to compute the brownian motion, we converted all forces parameters into arbitrary units relative to the temporal discretization (the higher the forces, the lower the time discretization). We used a set of parameters that combined both smooth simulation and computation efficiency. These parameters describe the physical environment in which the experiments are conducted. Although they can be modified by the users, they will remain constant for all the experiments. This results in the following parameters: strength max: 500; torque max: 500.

The sizes of the elements are also relative. They are chosen in order to fill sufficiently the cell nucleus while allowing free protein movement. This results in the following parameters in all the simulation shown: cell radius: 50; first protein radius: 1; second protein radius: 3. Note that the arbitrary units used for the forces are related to the size parameters (the movement of the proteins and of the multi-protein structures are related to the force and torque on the basis of their mass and kinetic momentum, i.e. their volume and shape).

Once the movement and collision are set and resolved, each protein in contact with another one will check its sticky position. That is each protein in contact will check the random value for the COS which allows to decide if the pair of proteins considered will stick or not, or remain stuck or not. Thus the sticking period for one given protein follows a geometric law of parameter COS: COS^t ^(1-COS), that gives the probability to stay stuck for t time points.

### FLIP-like experiments

Given the random movement of proteins, the "bleaching zone" is modeled through a probability value that a given single protein becomes bleached. This probability was set to 0.01 in the experiments shown. The same probability was applied to all proteins whatever their type.

**Figure 5:**
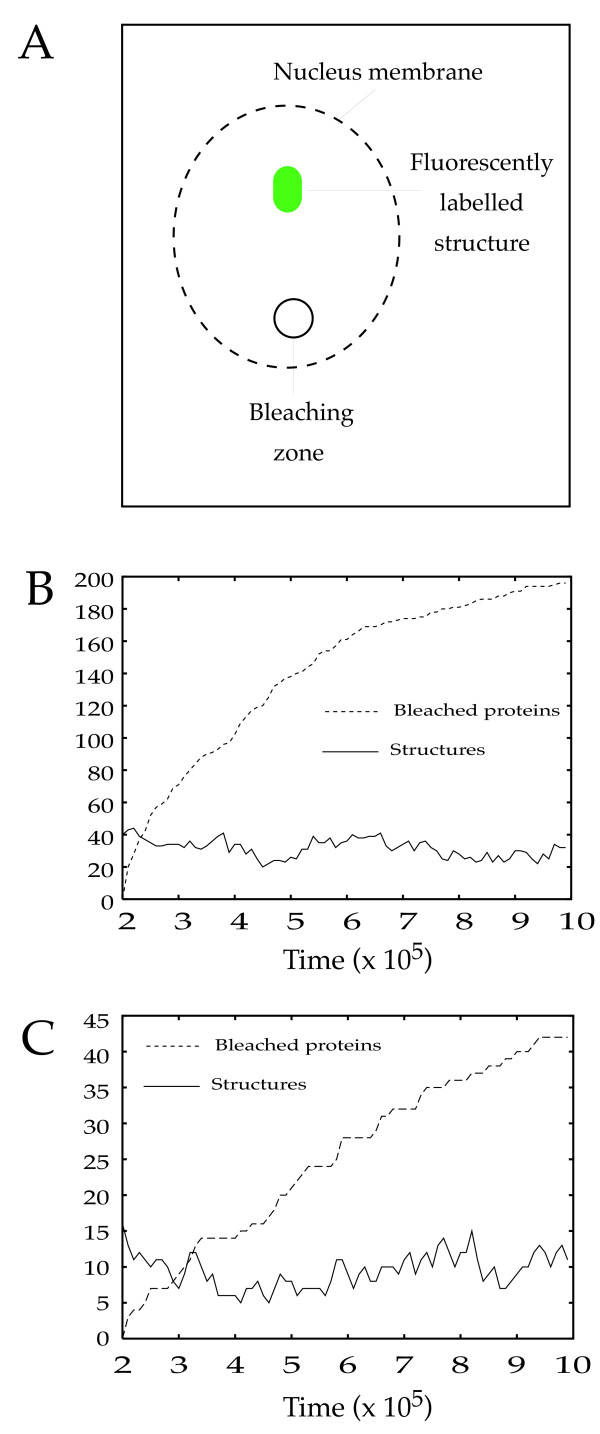
A virtual FLIP experiment. In A, the principle of the biological FLIP experiment is shown. One given, labeled protein, that participates in one given biological structure (shown in green, since it is fluorescent at the beginning of the experiment) is bleached whenever it passes through a given region of the nucleus (Bleaching Zone, a region of the nucleus where the laser is turned to the bleaching mode). The overall fluorescence of the structure it then studied as a function of time. If the protein moves freely out of the structure and into the cytoplasm, then it will at some point passes through the bleaching zone, be bleached, and by random movement be incorporated again in the structure,. The overall fluorescence of the structure will therefore decrease with time. B and C: Result of two individual *in silico *FLIP experiments. The following parameters were used: Protein number: 216; Number ratio: 0.5; Size ratio: 3 and COS = 0.9999 for the experiment shown in B and COS = 0.99999 for the experiment shown in C. The bleaching probability value (see Implementation section) was set to 0.01. Those results were confirmed by 10 independent simulations that gave a very narrow range of output (not shown so that the behavior of one individual simulation can be easily seen).

3DSpi was first run for 20000 time steps using the indicated parameters (see legend to figure 5) in order to reach a "stable state". Then the bleaching was started by applying the 0.01 probability value to become bleached to individual proteins not engaged in any interaction (i.e not stuck to anyone). This is equivalent, since particles move at random, to decide that the bleaching zone would occupy 1% of the overall nuclear surface. Once bleached, the protein remains in this state for the rest of the simulation. It can nevertheless still be engaged in interactions with other proteins, on the very same basis as non-bleached proteins.

In all experiments we recorded the number of structures through time. In the FLIP-like experiment we also recorded the number of bleached proteins.

## Results

### Modeling methodology

3DSpi (3-dimensional Dynamic Simulator of Protein-Protein Interactions) is a multi-agent simulation software that has been developed to model the global structures that can emerge from sets of interacting proteins. Such an approach relies on a strict modeling methodology: the aim of the model is to observe, at a global level, structures that are not explicitly programmed in it. For this, we introduce elementary entities (here the proteins) whose behavior only depends on local interactions. This approach means that the multi-protein structures (here the nuclear bodies) are not explicitly introduced in the model. Thus, if they are observed, we can argue that the self-organizing hypothesis is sufficient to explain their emergence. Moreover, the simulation can also help to characterize the qualitative behavior of the multi-protein structures, thus giving important information to predict the behavior of the original *in vivo *nuclear structures. It is important to note that our aim is not to use 3DSpi to model detailed protein folding or structure. Therefore, our software is fundamentally different from classical folding software which model the precise structure of a small number of proteins. On the contrary, in 3DSpi, the protein model is very simple (proteins are isotropic spheres, see below), but the purpose of 3DSpi is to predict the spatial structures of assemblies of a very large number of molecules.

### Basic functions of 3Dspi

3DSpi enable us to compute the interactions of a large amount of autonomous agents (i.e. there is neither centralized decision process nor high level compartments that exchange materials). All the agents are 3-dimensional solid particles moving in a 3-dimensional space that are able to interact with each other locally. In the simulation proposed here, it is used to simulate two different types of proteins. Each of them is modeled by an "interaction volume" which is considered as an homogeneous, isotropic, sphere. The two protein families differ by their size. Thus they move differently – the larger proteins move slower – and they fill the nucleus space differently. Therefore, the relative proportion of the two protein families may influence the system behavior.

The program requires four values to be defined as input for describing the biological system (plus fixed parameters describing the physical world, see Implementation section):

1. The total number of proteins occupying the "nucleus" space;

2. The ratio in number between the two sorts of proteins. If this value is equal to 0.5, then the same number of each protein species is used.

3. The respective size of the diameter of the two proteins types. If that value is set to 1, then this will results in simulating two proteins with the same size; if that value is set to 3, then this will results in a type of protein 3 times larger in diameter than the other.

4. The Coefficient Of Stickiness (COS), which is the probability, at each time point, that a protein attached to another protein will stay attached to it during the next time step (see Implementation section). The COS can be seen as a very macroscopic consequence of the folding properties of the proteins that sums the affinity of one protein species to another.

The program starts with a random distribution of the proteins. Then every protein is induced to move at random, until it hits another one. The probability that colliding proteins will bind to each other is then calculated as a function of the user-defined COS.

**Figure 1:**
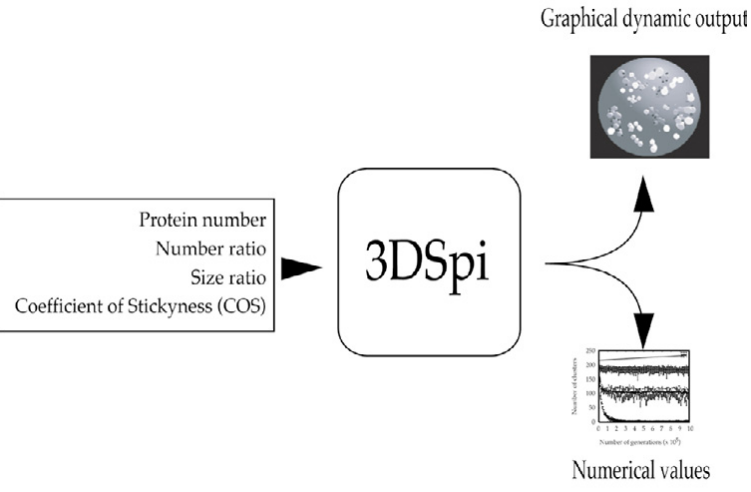
Schematic view of 3DSpi. Starting from local protein parameters (COS, protein size, ...), 3DSpi computes the proteins interactions and provides two different outputs: Video output and numerical data.

**Figure 4:**
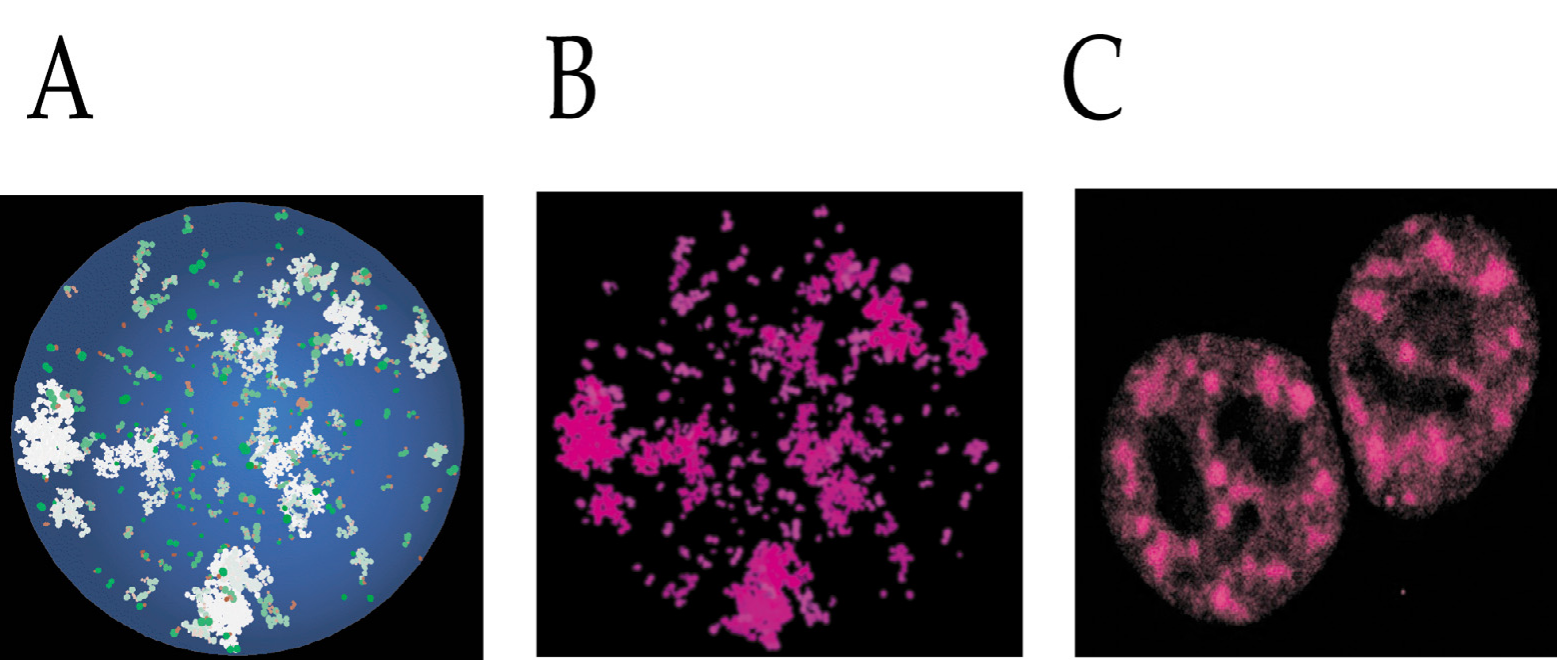
Images observed using 3DSpi. A: Screen shot of 3DSpi. The following parameters were used: Protein number: 3375; Number ratio: 0.5; Size ratio: 2; COS: 0.99999. The final state of the system is shown. B: The original image was treated with Adobe^® ^Photoshop^®^, in order to change the colors. C: A real life picture of speckles (reprinted with permission from SCIENCE [1]).

The program runs for a fixed number of one million time steps. Each run can be followed in real-time through a graphic interface that displays a 3-dimensional view of the system (filmed sequences can be obtained by contacting the authors; Figure 4A shows a screen shot). Various numerical values can be recorded during the run including the number of protein structures and their size. In 3DSpi, a "structure" is an *observed *structure of any size, from one protein to any number of proteins that are bound together. We did not define a structure as being more than one protein since multi-protein structures are not explicitly (i.e. *a priori*) introduced in the model. Both visual and numerical outputs are computed in real time. On one hand the videos enable the biologist to understand the behavior of the system and to propose hypotheses. On the other hand numerical data are mandatory to statistically validate these hypotheses and to analyze the self-organization behavior of the system precisely (figure 1).

### Generation of structures as a function of COS

**Figure 2:**
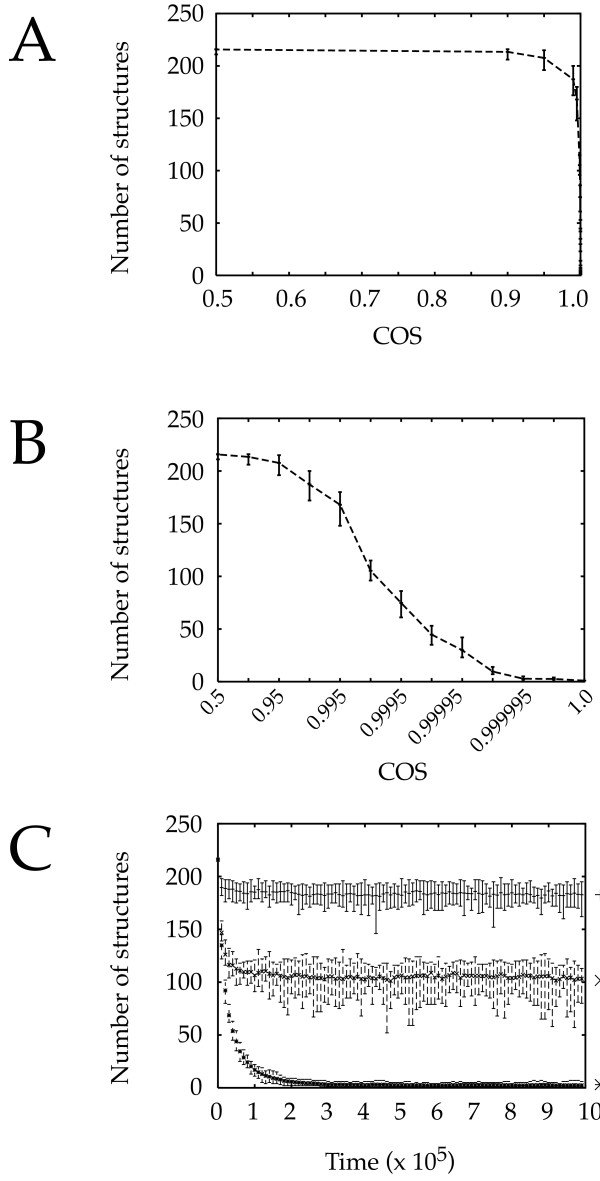
Number of structures generated using 3DSpi. A and B: Number of structures generated as a function of the COS value. The following parameters were used: Protein number: 216; Number ratio: 0.5; Size ratio: 3. The program was run for 10^6 ^time points. In B an enlargement of the right part of the figure in A is shown, on a semi-logarithmic scale. The mean observed during the last 50000 time points in one simulation is shown. The bar indicates the minimum and maximum value observed. C: Number of structures generated as a function of time using three different COS values (see the right part of the picture). The mean observed on 20 independent simulations is shown, and the bar indicates the minimum and maximum value observed in all of those simulations. Protein number: 216; Number ratio: 0.5; Size ratio: 3.

We examined how the structures evolved as a function of the COS value (Figure 2A and 2B). The behavior of the system can be predicted easily for two extreme values. With a COS value of 0 no protein binds to any other (i.e. all the "structures" are made of only one protein). Thus the observed number of structures equals the number of seeded proteins (see Implementation section for details). In the other hand, if the COS has a value of 1, all of the proteins will ultimately be bound to each other, resulting in only one very large "structure". These extreme cases are correctly modeled by our system (see Figure 2A).

There is a large interval of COS values (between 0 and 0.9) for which nothing happens. In that interval, although proteins collide and bind to each other, these interactions are too transient to generate any large stable structure. However for values comprised between 0.9 and 1.0, an exponentially increasing tendency to form structures is observed (Figure 2B). In this very narrow range the number of structures is a direct function of the COS. We verified that a very similar behavior of the system was observed for two other protein size ratio (size ratio of 1 and 4, not shown) and therefore that this phase transition was a robust behavior of our system.

Moreover, as far as the number of structures is concerned, the behavior of the system is highly reproducible: the extreme values (minimum and maximum number of structures for the different runs) observed during the last 20000 time steps (i.e. after the transient period) are very close to the mean. This suggests that the system is highly robust and generates a predictable dependency on the COS. In order to assess this statement, we ran 20 different simulations, for 3 different COS values, and we recorded the number of structures at each time step (Figure 2C). Two things were readily apparent:

1. The system reaches its equilibrium very quickly. Indeed in the worst case there is no significant change after 2.10^5 ^time steps.

2. The number of observed multi-protein structures is a function of the COS value. This means that the number of structures is independent of a particular stochastic run and therefore an invariant of the topology and of local behavior parameters (COS).

**Figure 3:**
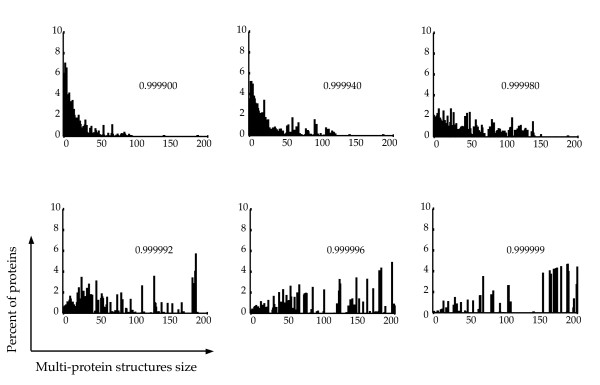
Size of the structures generated using 3DSpi. The percentage of proteins belonging to the various size structures is shown, ranging from 1 to 216, as a function of a COS value ranging from 0.999900 to 0.999999. The program was run for 10^6 ^time points. The mean observed for the last 10000 time points on 40 different simulations is shown.

We next analyzed the size of the multi-protein structures formed. In order to do this, we plotted the repartition of the proteins according to the size of the structure they belong to, for various values of COS (Figure 3). As expected the majority of proteins move from small structures to large ones when the COS increases. Interestingly, at high COS values, a dynamic equilibrium occurs between two groups of structures, small ones and large ones, without structures of intermediate size (see Figure 3, COS = 0.999999). This is characteristic of a phase transition in which small local differences can have a large impact on the global behavior. This indicates that one observes both the emergence of complex structures and the existence of complex interactions between these structures.

Altogether our data demonstrates that as the COS value increases, the system shifts from a state characterized by numerous small structures toward a system mainly composed of a small number of large structures.

### Ability of 3DSpi to mimic biological structures

3DSpi was initially intended to simulate nuclear bodies. Its ability to generate body-like structures was a necessary step toward its validation. We reasoned that in order to generate realistic data, one should approximate the real life observation conditions. For this, we initiated a 3DSpi run with a very large number of agents (of each sort). This resulted in a screen shot of a 3DSpi simulation (Figure 4A). Using an image processing software we modified the initial colors. This generated an image (Figure 4B) that is very strikingly similar to a real-life image of nuclear structures called speckles (Figure 4C). Speckles or splicing factors compartments are known to be dynamic structures, and both their protein and RNA-protein components can cycle continuously between speckles and other nuclear locations [15]. At a very macroscopic level, 3DSpi can therefore generate images of dynamic structures closely resembling those observed using confocal microscopy.

### Evidence that the structures generated by 3DSpi are dynamic

The most convincing way to demonstrate that proteins are continuously exchanged between a given nuclear structure and the nucleoplasm is called Fluorescence Loss Induced by Photobleaching (FLIP; [16]). In these experiments, a protein that participates in an observable structure is fluorescently labeled. The resulting fluorescent structure is visualized while a beam set to bleaching mode is used to photobleach a portion of the nucleoplasm (Figure 5A). Photobleaching consists in switching off the fluorescence associated with the protein without destroying the protein itself: the protein is still there and active but it cannot be detected by the confocal microscope anymore.

In the case of the transcriptional complex formed by the glucocorticoid receptor (GR), the FLIP approach demonstrated a rapid decrease in the fluorescence of the GR-containing complexes bound to DNA [16]. This demonstrated that the GR transcriptional complex was continuously exchanging individual GR molecules at a high rate with the nucleoplasm.

We decided to apply this FLIP approach to 3DSpi-generated multi-protein structures. For this, we decided to "bleach" individual proteins not engaged in an interaction, by applying a probabilistic bleaching value for the isolated proteins. It is obvious that this will underestimate the real bleaching since for example proteins dimers can pass through the bleaching zone in a real experiment, but not using our bleaching strategy. We nevertheless feel this is sufficient for probing the extent to which large structures are composed of particles that are continuously exchanged with the nucleoplasm.

We therefore followed the number of structures as well as the number of bleached proteins (Figure 5B and 5C) for two COS values (below and above the phase transition values). It was immediately apparent that for both simulations, the number of bleached proteins increased steadily during the course of the experiment while the number of structures remains stable. This thereby demonstrates that proteins are indeed continuously exchanged to and from the structures and thus confirms the dynamic nature of the multi-protein structures that could intuitively be deduced from the visual system's observation.

The dynamic properties shown by this experiment is a very fundamental result since it shows that, though at a macroscopic level the system behavior is different for different COS values, at a microscopic scale the protein behavior is similar. However, the dynamic of the exchange was clearly influenced by the value of COS. When the lowest COS value was investigated, at the end of the simulation, virtually all of the proteins were bleached, whereas with a larger COS value only about 20% of the proteins have been bleached at the end of the simulation period. In the case where a high COS value is used, it might seems surprising that even deeply buried proteins can be bleached. We explored this issue using the video output of 3DSpi. We observed large structures happen to "break in two" thereby exposing their inner core and exposing the proteins from the inside of the structure. The biological relevance of such a phenomenon needs to be assessed.

## Conclusion

We have developed a program called 3DSpi that simulates the behavior of 3-dimensional solid particles, moving at random in a 3-dimensional space, colliding, and binding to each other as a function of a probabilistic value called Coefficient Of Stickiness (COS). Dynamic multi-particles structures appear only for a narrow range of COS values. Within that range, the behavior of 3DSpi, although intrinsically stochastic, and therefore noisy, was shown to be very robust, as assessed by the predictable number of emerging structures, and by the short period of time required to reach a dynamic equilibrium. Moreover, a phase transition occurs to give two distinct distributions of structures. This non-linear feature of the system generated by 3DSpi can be used to model several of the non-linear phenomena found in biological systems. In addition the structures generated by 3DSpi are very realistic as they appear to resemble real life structures. Furthermore, the use of an *in silico *FLIP-like technique confirmed that the behavior of our model was compatible with the existing data regarding the dynamic nature of cellular substructures [1].

One other published study using MAS to model molecular structures has been conducted in a virtual 2-dimensional space [4]. In our preliminary experiments, we found that 2-dimensional versions of our software were inefficient for generating biologically relevant structures (data not shown). Since biological phenomena occur in a 3-dimensional space, it is therefore essential that studies conducted on spatial structures are performed in a realistic 3-dimensional space. Furthermore, a different modeling strategy was used in [4], in which high-level scenario were explicitly introduced. A more recent 3-dimensional version of a program called HSIM has been proposed [9], that uses rules for encoding the relations between the proteins. Unfortunately, a quantitative analysis of HSIM behavior has not been published, and its availability not publicized, therefore precluding a direct comparison with our 3DSpi model.

In our study, we used a modeling methodology that was strictly designed so as not to encode explicitly the structures we wished to study. As such it generates a surprising consequence (at least in contrast with more classical modeling tools): since the structures are not explicitly programmed-in, the 3DSpi software cannot itself provide their global parameters. In other words, it can not describe the parameters of structures that "don't' exist" at its own modeling level. Therefore we developed an appropriate independent observation tool, just as biologists do while observing real structures.

One of the indirect results of our work is to ask a fundamental – but rarely evoked – question: what is a structure? Multi-Agents System models and 3DSpi can help answering such a question. Indeed, in our system structures are no longer considered as fixed bodies whose components are clearly identified. They are the product of collective protein behaviors that are self-regulated, i.e. do not depend upon a "master" pattern. This self-regulation is the consequence of two opposite (and competing) trends inside the system. On the one hand, the entropy tends to increase disorder and create free proteins. On the other hand the stickiness moves the system toward a more ordered state and ultimately toward a unique and extremely stable large structure. The zone of interest lies around where these two opposite forces attain a dynamic equilibrium – a zone we termed as phase transition. This is in the latter that we found most of the non trivial structures, like a split occurring between two groups of structures, small ones and large ones, without structures of intermediate size.

Our initial aim was to test the possibility that very simple local rules are sufficient, when expressed in a physically relevant model, to generate sophisticated large structures. In particular, we wanted to determine "whether nuclear organization can be reproduced in silico assuming the constraints of self-organizing systems" [1]. We have demonstrated that this does occur. Our modeling results strongly favor the hypothesis that the appearance of large multi-molecular structures does not require sophisticated scaffolding. A transient non-specific protein-protein interaction is sufficient for generating large multi-molecular complexes, provided that affinity or stickiness of protein species lies within the proper range. Of course, the COS value cannot be seen as identical to an affinity or avidity value. It nevertheless remains quite conceivable that a COS-like interaction value can be evaluated using a composite function of biochemically-determined binding parameters. The mathematical approaches to FRAP modeling [17,18] should be helpful in estimating such a parameter from real life observations.

One should stress that we have demonstrated the *possibility *that such structure emerge by self-organisation, but of course this does not *demonstrate *that this is the case for real life structures. It is our belief that 3DSpi might be helpful in making predictions about how such a self-organizing system might behave that ultimately could be tested experimentally in living systems. However, predictions could only be made in a more biologically sound version of 3DSpi since the present status of 3DSpi model suffers from a number of limitations. Those limitations include the low number of proteins, the limited number of different proteins species and the uniform interaction pattern (i.e. all interactions are the same, irrespective of the proteins species).

In order to increase the biological relevance of our simulations, proteins should not be considered as homogeneous isotropic spheres. For this we are currently developing an XML-based tool that will allow to construct sophisticated proteins containing multiples domains, with each domain having its own set of interaction rules. To some extent one might envision to derive from real life 3-dimensional protein structures a simplified, but realistic, spatial version that can be modeled using 3DSpi. Using a larger amount of proteins, more different protein species and more sophisticated 3-dimensional versions of proteins, will inevitably require much longer calculation time. This problem might be solved through parallel computation on a computer cluster, a project in progress in our laboratories.

Furthermore, it would be very interesting to simulate protein synthesis and degradation rates, in order to assess the impact in variations of those parameters upon the global behavior of the system.

Another improvement may consist of adding consequences to the binding. For example, one protein could be "activated" (i.e. would now be able to interact with another one) only after having been bound to a third partner. This should result in modeling a signaling pathway, based on probabilistic interactions and random displacement. It would be very interesting to analyze the ability of such a pathway to carry a signal from the outside of a "cell" into its nucleus, as simulated by two encased spheres.

Finally, it is our belief that, as exemplified in the present work for spatially constrained structures. SMA-based modeling will proved to be a precious tool for tomorrow's biologists. Although this early version is restricted in its purposes, we are confident that later versions of 3DSpi will allow to model any type of biological structures, whether modules [19], or hyperstructures [7].

## Availability and requirements

OpenDE uses a GPL license and is available at . The OpenSPEAR library is also GPL and is available at . It is distributed freely with no online help or warranty. The 3DSpi program itself (3dspi_code.tar.gz) is also distributed under GPL license. It is available at . The Windows beta-testing implementations of OpenSPEAR and 3DSpi are available at . Linux OS versions of the 3DSpi libraries are available on request by mail to the authors. All further information (including software update) will be available through the following website: . A Windows stand-alone version of 3Dspi (3dspiInstallerv1.0.exe) is also available on the internet 

## Authors' contributions

H. S., F.P. and S. G. wrote the software. C.R. made most of the experiments described. G.B. and O.G. conceived the study, and participated in its design and coordination. O.G. wrote the initial draft. H.S., C.R., O.G. and G.B. participated in writing the final version of the manuscript. All authors read and approved the final manuscript.
